# pressuRe: an R package for analyzing and visualizing biomechanical pressure distribution data

**DOI:** 10.1038/s41598-023-44041-6

**Published:** 2023-10-05

**Authors:** Scott Telfer, Ellen Y. Li

**Affiliations:** 1 Department of Orthopaedics and Sports Medicine, University of Washington, 1959 NE Pacific St, Seattle, WA 98195 USA; 2 Center for Limb Loss and MoBility, VA Puget Sound Healthcare System, Seattle, WA USA; 3 Department of Mechanical Engineering, University of Washington, Seattle, WA USA

**Keywords:** Biomedical engineering, Musculoskeletal system

## Abstract

In many biomechanical analyses, the forces acting on a body during dynamic and static activities are often simplified as point loads. However, it is usually more accurate to characterize these forces as distributed loads, varying in magnitude and direction, over a given contact area. Evaluating these pressure distributions while they are applied to different parts of the body can provide effective insights for clinicians and researchers when studying health and disease conditions, for example when investigating the biomechanical factors that may lead to plantar ulceration in diabetic foot disease. At present, most processing and analysis for pressure data is performed using proprietary software, limiting reproducibility, transparency, and consistency across different studies. This paper describes an open-source software package, ‘pressuRe’, which is built in the freely available R statistical computing environment and is designed to process, analyze, and visualize pressure data collected on a range of different hardware systems in a standardized manner. We demonstrate the use of the package on pressure dataset from patients with diabetic foot disease, comparing pressure variables between those with longer and shorter durations of the disease. The results matched closely with those from commercially available software, and individuals with longer duration of diabetes were found to have higher forefoot pressures than those with shorter duration. By utilizing R’s powerful and openly available tools for statistical analysis and user customization, this package may be a useful tool for researchers and clinicians studying plantar pressures and other pressure sensor array based biomechanical measurements. With regular updates intended, this package allows for continued improvement and we welcome feedback and future contributions to extend its scope. In this article, we detail the package’s features and functionality.

## Introduction

The ability to measure pressure distributions across human-object and within-body interfaces is an important technique for researchers and clinicians looking to understand how tissues behave and interact with their environment. Biomechanical applications of pressure measurement are wide-ranging, such as examining the ergonomics of seat positioning^1^, describing joint loading^2^, and studying residual limb/prosthetic interfaces^3^. Most commonly, it is used to understand the interactions between the plantar surface of the foot and either the ground, shoe, or insole^4^. At the foot, the relationship between pressure variables and clinical or ergonomic outcomes is often multifactorial, and this is still an active area of research. For example, studies have identified significant correlates between variables derived from pressure measurements and outcomes such as pain and tissue damage^5,6^. Other research has shown that pressure data can be used to aid in the screening and assessment of pathological conditions as well as the prescription and design of interventions^7,8^.

To measure barefoot pressure distributions across the plantar surface of the foot, individuals are typically instructed to walk over a rigid plate containing an array of sensors. Alternatively, if interested in the pressure distribution between the foot and a shoe, flexible sheets embedded with sensors can be placed within a shoe to assess shod conditions. Both methods can require matrices of tens to hundreds of sensors that are generally based on resistive or capacitive technologies. Sampling frequencies vary depending on the application, with walking and running activities often captured at 50–100 Hz^4^. As a result, large amounts of data can be generated in a relatively short period of time, potentially making effective interpretation challenging^9^.

Beyond the amount of data produced, there is neither a standardized export format for pressure data nor complete transparency as to how each proprietary software package collects and analyzes them. Of note, a recent meta-analysis has shown that there are differences between the results reported for different data collection systems^10^. Across the methods for collecting barefoot and shod plantar pressure distributions, data collection systems use different sensor shapes, sizes, and types of arrays, along with capture rates and underlying technologies. Therefore, their “raw” data formats can differ substantially, making data transfer and generalization difficult. In addition, the ability to access archived data in the event of newly available processing techniques can be limited by expired licensing agreements. This lack of accessibility contrasts dramatically with other tools for the biomechanical assessment of gait and other activities (i.e. kinetic, kinematic and electromyographic measurements) where there is extensive freedom in terms of the ability to move the “raw” data across different software packages. This is in part due to standardized or semi-standardized formats like .c3d and .mot, which facilitate data sharing and support the reporting of reproducible analyses.

Another important issue is that the current segmentation of systems and lack of reporting standards have contributed to imprecise definition of many relevant pressure distribution variables. As pressure measurement literature has grown, new metrics have been developed while existing metrics have assumed different methods of calculation^4^. However, it is often unclear which version is being incorporated. For example, there are at least two different definitions of the commonly reported pressure time integral^11^, and ambiguous terms like “mean peak pressure” abound^12,13^, where it is often not clear whether the mean is spatial or temporal.

In recent years, as part of a greater move towards open and reproducible research, many journals have been encouraging authors to provide data and analysis code with the goal of allowing easier replication of scientific research. Indeed, in many cases this is now a condition before a manuscript will be considered for publication^14^. The use of open-source software helps to facilitate easy and unconstrained code sharing to ensure reproducibility of often increasingly complex processing and analysis pipelines. Such practices assist with error detection (and correction), and allow scientists to build on the work of their peers.

In this article we describe the features and functionality of pressuRe. This software package is an open-source tool for biomechanical pressure measurements and is built in the freely available R statistical computing language. The package aims to allow data from a range of hardware sources to be processed and analyzed in a standardized manner and provides a number of flexible functions that the user can build their analyses upon.

## Description

The pressuRe package is written in the R scientific computing language^15^, which is an open-source, functional language and environment for statistically exploring and visualizing datasets. Users can develop custom packages in R for a range of purposes and can easily disseminate these through the Comprehensive R Archive Network (CRAN). As a note, we recommend running the pressuRe package using the RStudio Integrated Development Environment^16^, which has a number of features that help to enhance the use of the package.

The description of the package’s functionality is primarily focused on dynamic plantar pressure measurements (both in-shoe and platform based). However, many of the functions are applicable to other pressure measurement situations, such as data collected at a prosthetic/body interface, and we plan to include additional tailored functions for these applications in the future.

The major functions of the pressuRe package, detailed in Table 1, aim to simplify the analysis of regional dynamic plantar pressure distribution data into three main steps, each of which are complemented by a range of options for visualization:

1. Import and format data from different systems into a standardized format.
2. Process data in preparation for analysis.
3. Analyze the data to determine the required pressure variables.

**Table 1:** Primary pressuRe functions (see package manual for further details).

Function	Description
Importing and formatting data	
load_emed()	Read and format data collected using Novel emed (“.lst”) in a standardized format
load_pedar()	Read and format data collected using Novel pedar (“.asc”) in a standardized format
load_pliance()	Read and format data collected using Novel pliance (“.asc”) in a standardized format
load_tekscan()	Read and format data collected using Tekscan systems (“.asf” or “.csv”) in a standardized format
load_footscan()	Read and format data collected using footscan system (“.xls”) in a standardized format
Processing data in preparation for analysis	
select_steps()	Allow the user to interactively select steps/cycles from a trial and adding start/end event points for those selected
whole_pressure_curve()	Calculates whole foot curves for peak pressure, contact area, or force with option to plot
pressure_interp()	Interpolates pressure data to a set number of frames
auto_detect_side()	Attempts to automatically detect if barefoot pressure data is from right or left foot
cop()	Calculate center of pressure
create_mask_auto()	Automatically generates masks for pressure data. User can select from a list of available masks
create_mask_manual()	Allow the user to interactively generate regional mask by clicking points to outline regions or select sensors
edit_mask()	Allow the user to interactively select and edit the position of mask vertices
Analyze the data to determine the required pressure variables	
cpei()	Calculate Center of Pressure Excursion Index
mask_analysis()	Calculate a variety of pressure, force, and contact area metrics for each regional mask. We refer the user to the source code for the base calculations, however, for example peak pressure sensor is $\mathrm{max}({x}_{i,j})$ (1) where x is the data matrix of pressure data for the selected sensors in the region, and pressure time integral as defined by Melai is $FTI= \int F \times \Delta t$ (2) where ∫F × Δt is the integral of force over time $PTI=\frac{FTI}{A}$ (3) where FTI is calculated from Eq. (2) and A is the contact area of the region
Data visualization	
plot_pressure()	Generate visualizations of the pressure data with several options for customization
animate_pressure()	Generate an animated .gif of the pressure data with several option for customization

### Importing and formatting data

At the time of writing, pressuRe can accept data collected on emed® (novel GmbH, Munich, Germany), pedar^®^ (novel GmbH, Munich, Germany), I-Scan™ (Tekscan Inc., Norwood, MA, USA) F-Scan™ (Tekscan Inc., Norwood, MA, USA), and footscan (Materialize NV, Leuven, Belgium; formerly RSScan NV) systems, with support for others to be included in the near future. The resulting data is stored in a standardized list object consisting of the pressure data, pressure system type, sensor areas, sampling speed, regional mask shapes, event time points, and sensor vertices. This format may be extended to include other relevant items in the future.

### Processing data in preparation for analysis

Functions are available for a range of processing and pre-processing steps including interpolating data and generating overall peak pressure, contact area, and force vectors. In addition, there are functions specific to barefoot data to help the user detect which foot the data comes from, calculate the center of pressure, and more.

It is important to note that data collected from a rigid plate generally captures a single step (although some larger plates allowing multiple steps to be captured in a single trial are now available, support for these will be added in the future). In-shoe sensors, in contrast, generally collect multiple steps in a single trial and thus require an additional processing step in pressuRe to identify the timing of individual steps. Through a semi-automatic step detection method, pressuRe includes a function that helps the user to identify “suitable” steps by giving options to remove initial steps and presenting overlaid force curves for potential steps.

Currently, the most commonly reported method for analyzing plantar pressure measurements utilizes a regional analysis. In this method, certain anatomical areas (i.e., rearfoot, midfoot, metatarsal heads (MTHs), toes) or combinations of said regions are “masked” and analyzed individually using variables such as peak pressure during stance. While there are limitations with this approach^17^, it remains widely used and is generally easy to interpret. Therefore, a number of pressuRe functions are available to facilitate this type of analysis.

Regional masks can be defined as percentages of the overall foot shape or by identification of anatomical landmarks. The pressuRe package includes functions to allow the user to, for example, manually define custom masking regions (create_mask_manual()), by selecting groups of sensors or drawing round regions of interest. Pressure footprints can also be applied automatically (create_mask_auto()) in this case applying predefined template masking schemes for shod data (“pedar_mask1”^13^, “pedar_mask2”^18^, and “pedar_mask3”^19^) and an automatic regional masking algorithm designed for barefoot plate data (this is a relatively simple implementation which may fail for certain pressure patterns and will undergo further developed in the future). Once defined, masks can be edited (edit_mask()) or additional ones can be created and added to the existing regional mask list, which are stored as a list in the fifth element of the pressure dataframe. The existing masking algorithms and templates aim to help standardize how regions of the foot are analyzed, while allowing for customization and user flexibility.

### Analyze the data to determine the required pressure variables

A variety of functions and metrics are included in the pressuRe package to analyze pressure distribution data across the entire plantar surface and within each mask region (Table 1). Options are available to include or remove sensors that are only partially covered by the mask in the analysis. The package also implements other pressure variables reported in the scientific literature like center of pressure excursion index^20^ and dynamic plantar loading index (DPLI, also known as regression factor (RF) index)^21^, which have been used to indicate characteristics like foot posture^22^ and abnormal foot function^23^. For regional analysis, certain metrics like pressure time integral have been calculated in a variety of ways, changing how data is meant to be interpreted. Thus, the pressuRe package includes both the pressure time integral defined in the novel software package and that defined by Melai et. al^11^ to increase reporting transparency and choice. Additional variables will be added in the future as the package matures.

### Data visualization

To increase user customization options and to make data assessment and analysis more intuitive, the pressuRe package includes a variety of data visualization functions (Table 1). Understanding the dense data that a plantar pressure measurement is typically composed of often requires a visual representation of changes in pressure over time. This could be the maximum or average pressure experienced in each area of the foot, or pressure at a certain time point. Beyond integrating these time-based visualization plots, pressuRe also allows the user to define the sensor colors and respective pressure ranges, as well as options to include sensel or footprint outlines. It also allows additional pressure static overlays such as the center of pressure path. These features provide both easy visualization for quality control checks during processing, but can also be exported as high resolution images for manuscripts and presentations. In addition, the package also allows users to generate and export a GIF animation of the pressure data for presentation purposes.

**Figure 1:**
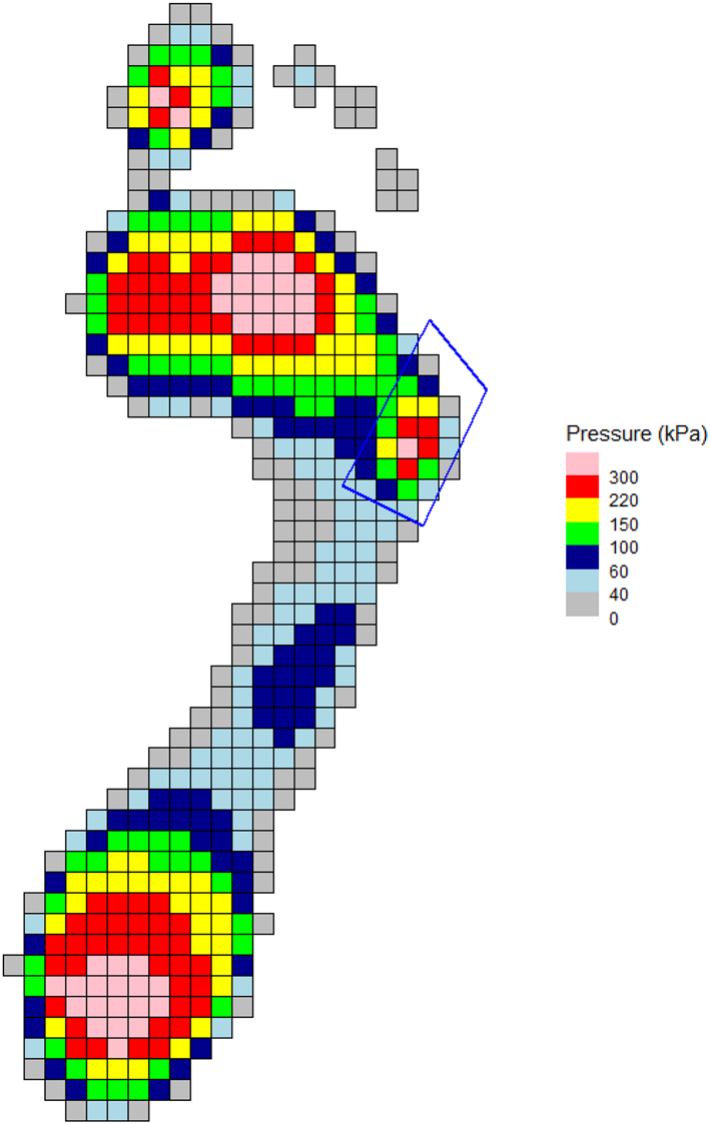
Custom mask interactively defined at the region of the 5th metatarsal head.

Data visualization and user interaction assists the user with controlling the quality of their data. For in-shoe or other “cyclic” datasets (i.e., those collected on Novel pedar^®^, Tekscan I-Scan™, and Tekscan F-Scan™), selecting the beginning and ending of a step is important for accurate data analysis. To streamline this process, the select_step() function first uses a user-defined force threshold to identify the beginning and end of steps. Then, it displays all identified steps and sequentially highlights the force curve of each step, asking the user to decide whether they should keep or discard the step from analysis. Other examples of interactive features can be found within the regional masking functions described earlier. When using these functions, a pressure distribution plot is displayed and the user can select vertices that define the border of regional masks, allowing for the addition of custom, non-template masks (Fig. 1). The user can also, in a similar manner, edit mask boundaries as necessary.

### Example workflow

Here we provide simple examples and descriptions of the pressuRe functionality. Example data are taken from a previous study^24^. Although we cover more options here, in general we have worked on the principle that a file that represents a single step of data can be processed in 3–4 lines of code, going from raw data to an output variable, such as the peak value within a masked region. As the package matures, we plan to make vignettes available to demonstrate example approaches for processing large data sets along with simple statistical analyses.

If using for the first time, the package needs to be installed:

- > install.packages(“pressuRe”)

Then, the package can be loaded:

- > library(“pressuRe”)

Next, import your pressure distribution data (in this case from the emed system):

- > emed_data <-load_emed(“C:/User/data/pressure_data_example.lst”)

When working with datasets involving multiple trials, it is often useful to normalize each step to 101 frames so that the data can be described as a percentage of stance and multiples steps can be averaged:

- > emed_data <-pressure_interp(pressure_data = emed_data, interp_to = 101)

**Figure 2:**
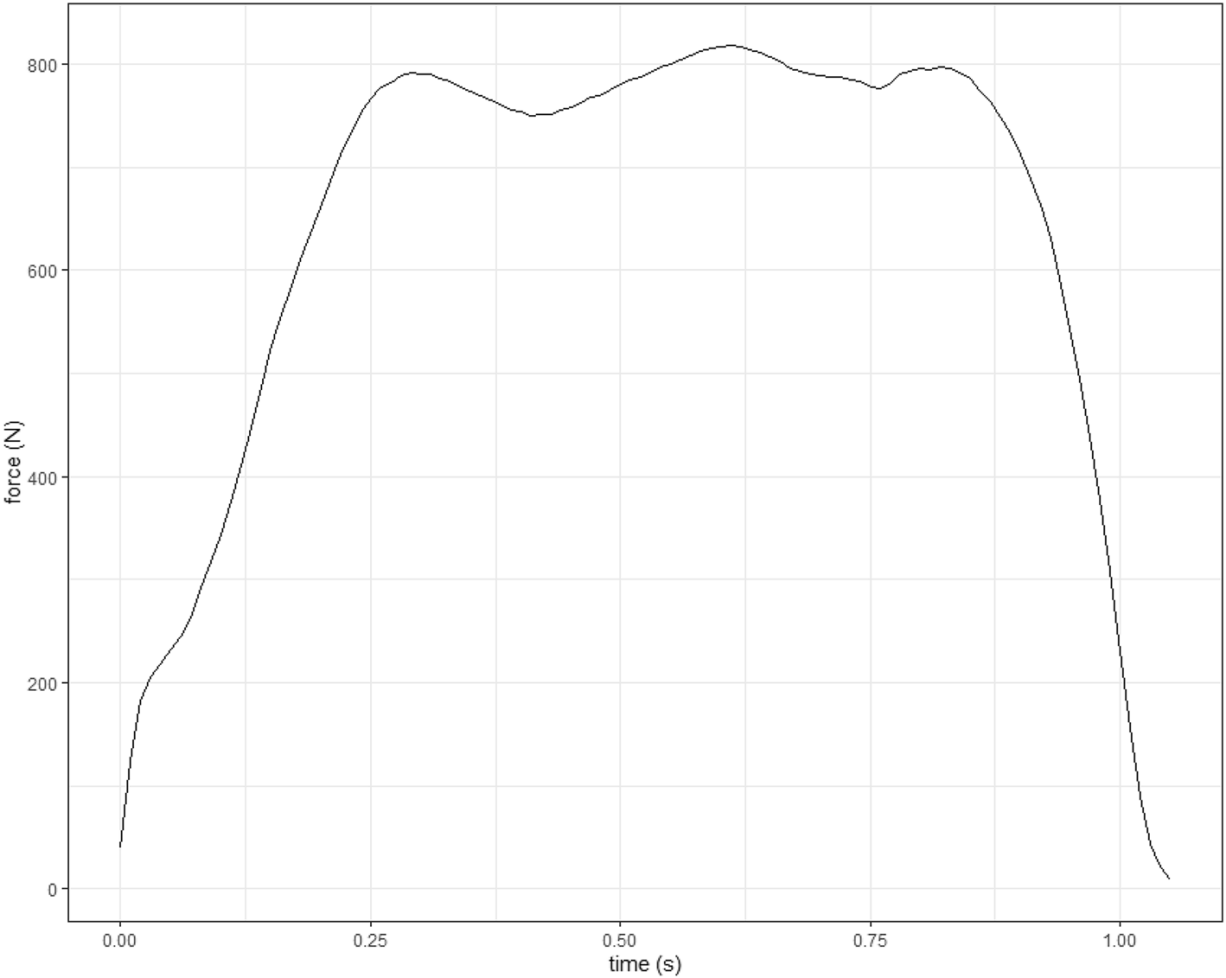
Force (summed across all sensors) vs time curve generated for pressure data trial of walking gait.

To quickly visualize your data and check there are no obvious issues, plot the force curve (Fig. 2). This is calculated by summing the pressure values multiplied by the area for each timepoint:

- > whole_pressure_plot(pressure_data = emed_data, variable = “force”)

**Figure 3:**
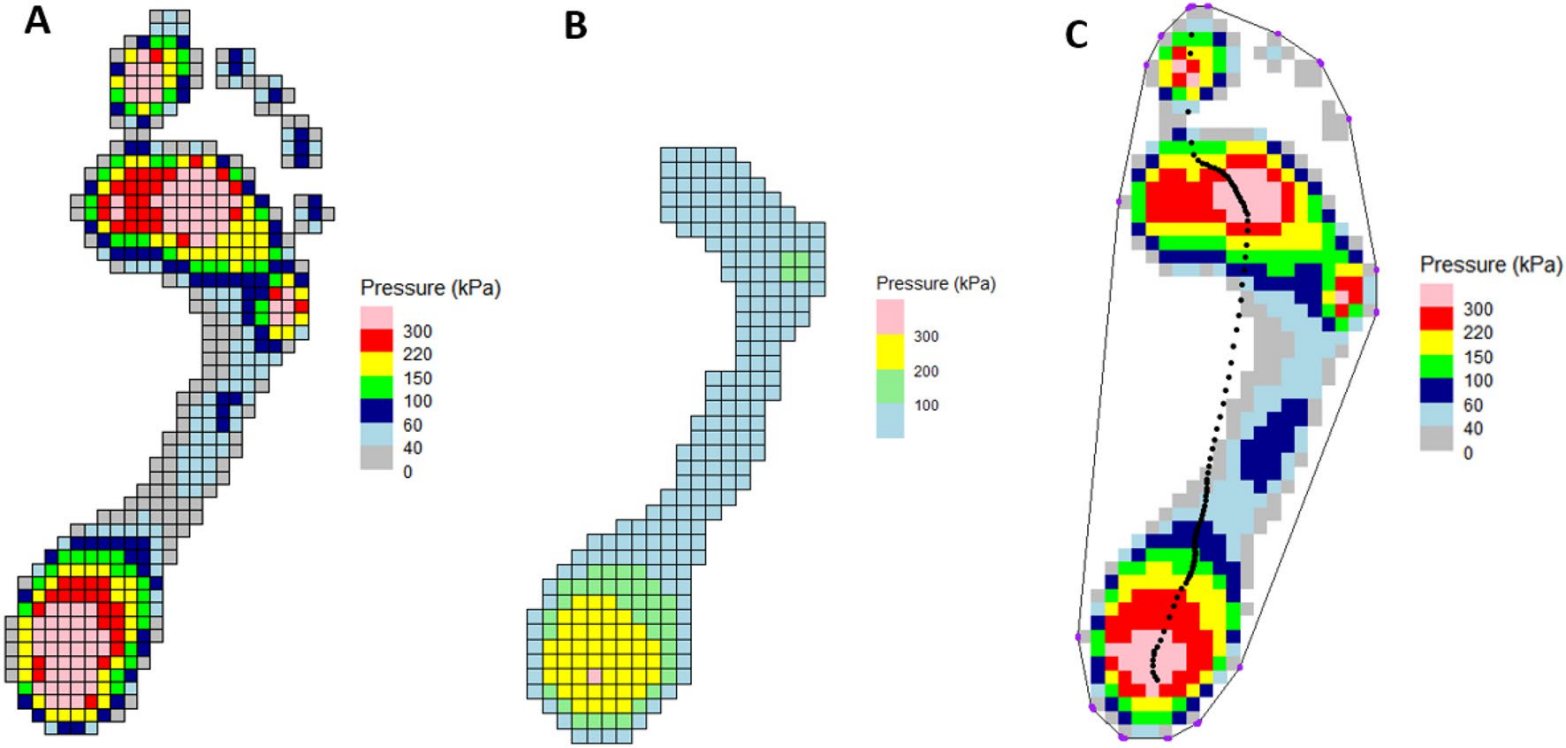
Examples of plotting options for pressure data. (**A**) Footprint of maximum values for each sensor with a standard color scheme and sensors outlined; (**B**) single frame of pressure measurement with custom color scheme; (**C**) maximum pressure footprint with center of pressure plotted, convex hull outline of footprint, and sensor outlines removed.

It may also be helpful to visualize the peak pressure at each sensel, creating a pressure distribution map of the foot. The simplest way to achieve this is shown below, which plots the maximum recorded pressure for each sensel using color mapping based on the typical “novel” pressure color and pressure range (Fig. 3A):

- > plot_pressure(pressure_data = emed_data, variable = "max")

Additional customization can be used to plot the recorded pressure for each sensor at a specific time frame (in this case frame 20) with a custom pressure range and colors: light blue (0–100), light green (101–200), yellow (201–300), and pink (301+) (Fig. 3B):

- > plot_pressure(pressure_data = emed_data, variable = "frame", frame = 20, plot_colors = "custom", break_values = c(100, 200, 300), break_colors = c(“light_blue”, “light_green”, “yellow”, “pink”))

Another example is plotting the maximum recorded pressure for each sensel with a center of pressure overlay, a footprint outline, but no individual sensor outline, and no legend (Fig. 3C):

- > plot_pressure(pressure_data = emed_data, variable = "max", plot_COP = TRUE, plot_outline = TRUE, plot_colors = "default", sensor_outline = FALSE, legend = FALSE)

If visualizing the pressure distribution map at each timepoint in a step is useful, it is possible to export an animated .gif of the pressure distribution data (note that depending on the size of the trial, this may take several minutes to complete):

- > animate_pressure(pressure_data = emed_data, plot_colors = "default", fps = 100, dpi = 96, file_name = “example_pressure_animation.gif”)

**Figure 4:**
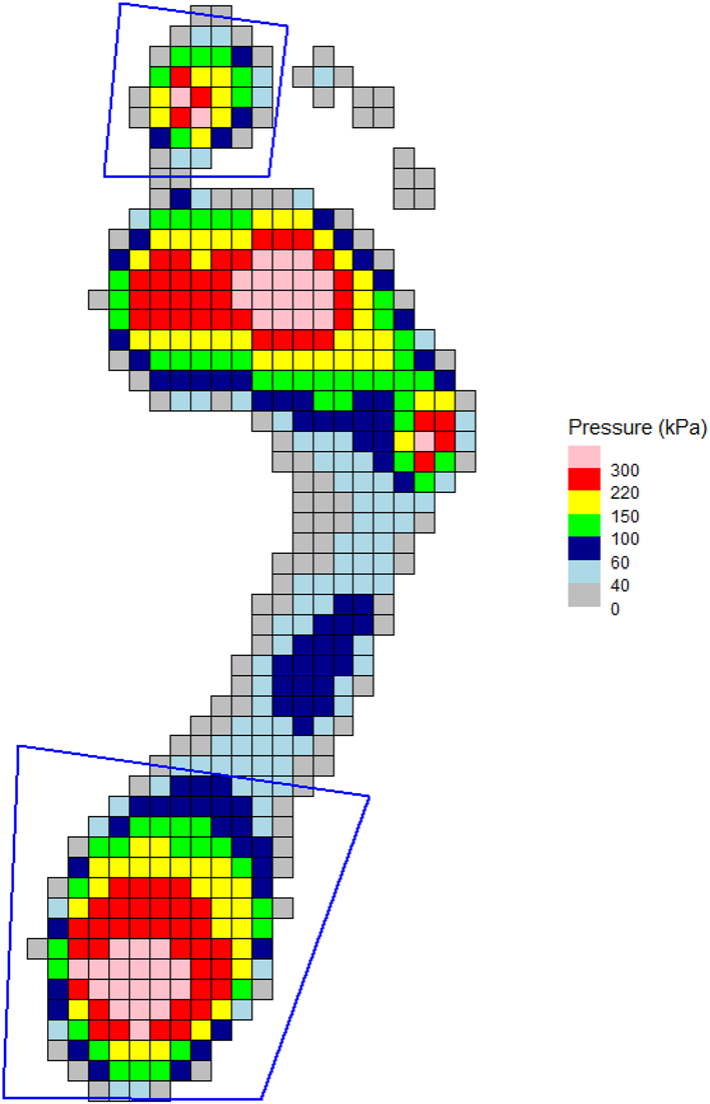
Regional masks manually created for heel and hallux.

Extracting pressure metrics at certain anatomical regions of the foot can also be advantageous when looking to analyze plantar pressure distributions. These can be manually defined, as shown here for the heel and hallux (Fig. 4). This is achieved by setting the masking parameters to use 4 vertices, 2 masks, with the mask names “hallux_mask” and “heel_mask.” The user interactively defines the four vertices of the hallux mask by clicking on the graphic, starting with the top left corner, then repeats for the heel mask:

- > create_mask_manual(pressure_data = emed_data, mask_definition = “by_vertices”, n_masks = 2, n_verts = 4, mask_names = c("hallux_mask", “heel_mask”))

**Figure 5:**
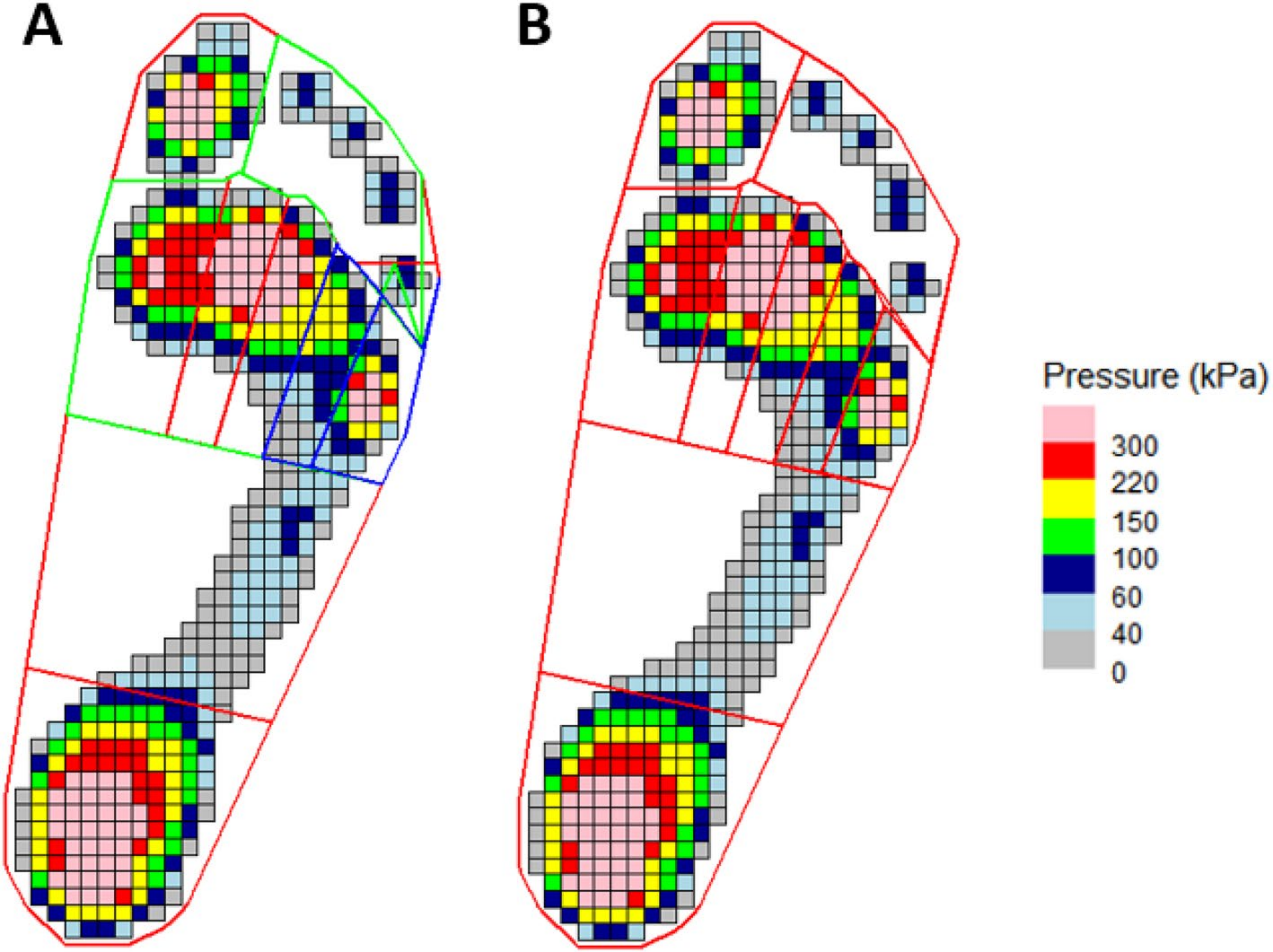
Example of masking scheme created using automask function with MT5 mask before (**A**) and after (**B**) adjustment.

For barefoot data, to define regions in the foot, an automatic algorithm is available that applies a standard masking scheme to the footprint. This attempts to produce 10 regional masks. Given the developmental nature of this function at the time of writing, we recommend always plotting the masks for visual confirmation (Fig. 5):

- > create_mask_auto(pressure_data = emed_data, masking_scheme = “automask_novel”, foot_side = "auto", plot = TRUE)

If a mask is unsatisfactory, it can be interactively edited. The user defines which mask and how many points need to be corrected, clicks on the required point to select, then clicks again for the new position (Fig. 5B).

- > edit_mask(pressure_data = emed_data, n_edit = 1, edit_list = c(“MT5_mask”))

**Figure 6:**
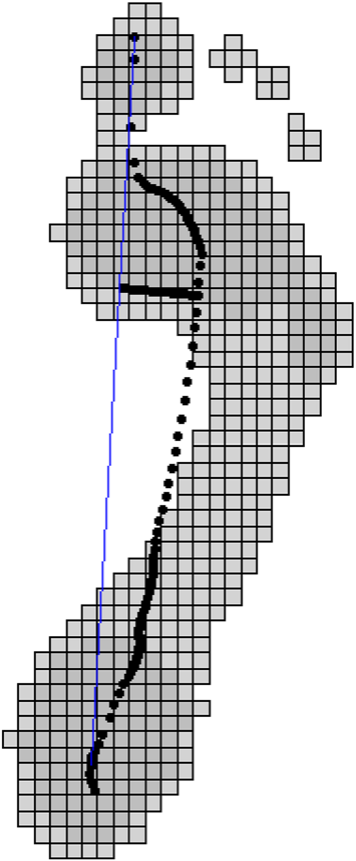
CPEI image. The center of pressure (dotted line) is plotted over the pressure image and a construction line marking its medial border is shown in blue. The distance between the center of pressure line and the construction line at the first anterior trisection of the foot is shown as a solid black line and the CPEI is calculated as the the ratio between the length of this line and the forefoot width.

The center of pressure excursion index can be used to understand deviation in center pressure from an expected path defined by the heel and third metatarsal head. To extract this value and visualize the deviation overlayed on a pressure image (Fig. 6):

- > cpei(pressure_data = emed_data)

**Figure 7:**
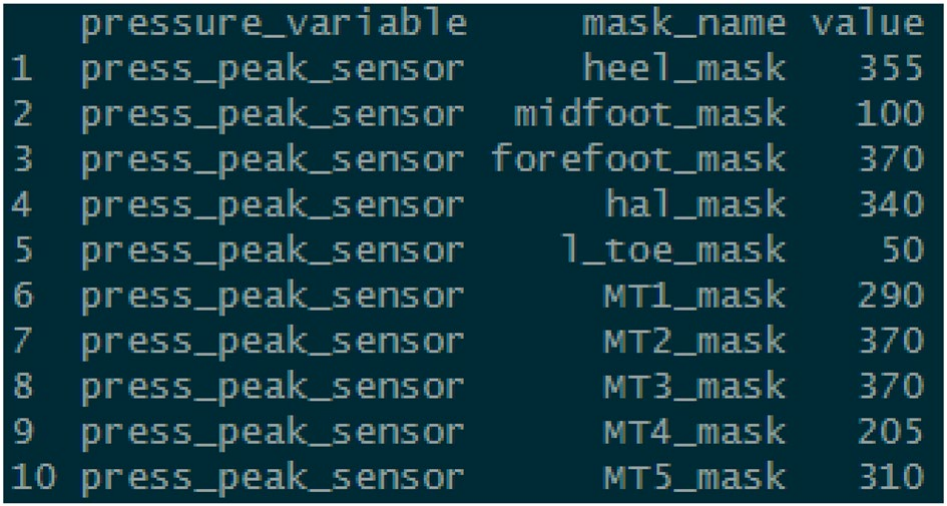
Pressure output results for regional masks.

A number of pressure variables can be calculated for each regional mask (Fig. 7). For the specific calculation used in each case, we would refer the user to the source code:

- > mask_analysis(pressure_data = emed_data, variable = "press_peak_sensor")

Additional examples for other functions can be accessed in the pressuRe help files.

### Example on clinical dataset

#### Design

As a demonstration of processing data using the pressuRe package, we used plantar pressure data collected during a previous study^24^ to assess if individuals with a longer duration of diabetes have associated changes in plantar pressures. Previous work has shown that this may be the case, however this was limited to peak plantar pressures^25^. In this analysis, we explore other pressure variables relationship to disease duration. The complete processing code demonstrating how to perform this type of analysis is provided in the supplementary material S1. Data was also assessed using commercially available software to ensure validity of the results.

#### Data

The pressure measurement data consisted of barefoot walking (5 trials each for left and right foot) for 16 participants with diabetic neuropathy. Data were collected using the emed^®^ system (novel GmbH, Munich, Germany) at self-selected walking speeds. Further details about the participants and data collection procedures can be found in the original paper.

#### Processing and analysis

We calculated peak pressure (highest value read by any sensor within region, averaged across steps), pressure time integral (peak pressure at each timepoint during step multiplied by time), and Dynamic Plantar Loading Index (DPLI; a dimensionless measure of the fit between time series peak pressures and a normal distribution) for the forefoot and hindfoot. We also calculated peak pressure and pressure time integrals using commercial software (automask, novel, GmbH, Munich, Germany) and compared these to the results from pressuRe. As a simple demonstration of using the statistical tools found in R, we performed an analysis of the calculated variables comparing participants with shorter duration disease history (defined as < 12 years) to those with longer (> 12 years) using a Mann–Whitney U test (α = 0.05). A linear regression model was also calculated, with disease duration treated as a continuous variable.

#### Results and discussion

**Figure 8:**
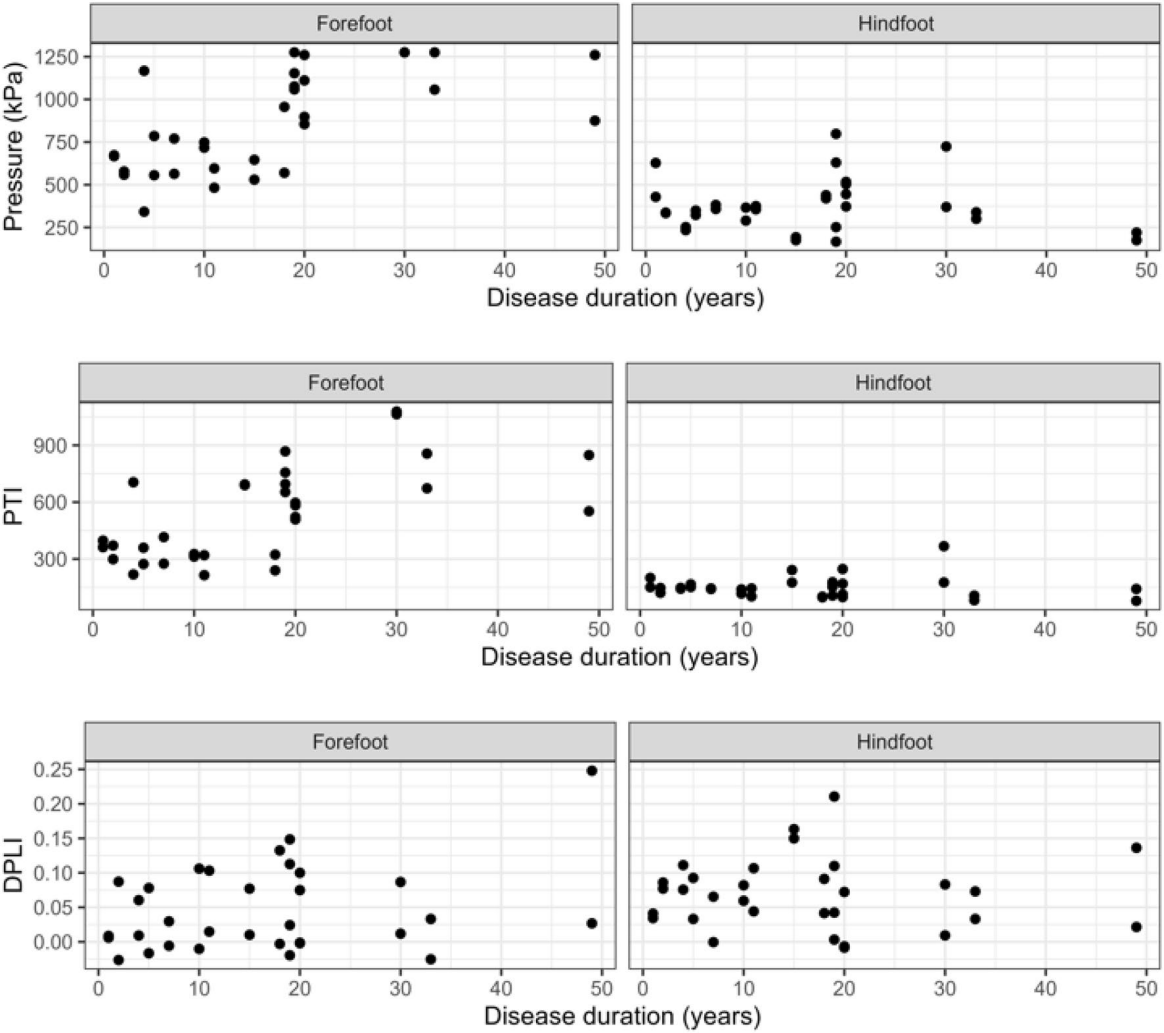
Effect of diabetes disease duration on pressure variables. Top row: peak pressure; middle row: pressure time integral; bottom row: DPLI.

Minimal differences were found between the results from pressuRe and commercially available software for the peak pressure and pressure time integral values (RMS errors < 1%). Any small differences were found to be due to minor variations in the mask definitions between software. Individuals with longer duration of diabetes were found to have higher forefoot pressures than those with shorter duration (Table 2). This was confirmed (Fig. 8) when disease duration was treated as a continuous variable (peak pressure forefoot: *p* < 0.001; pressure time integral forefoot: *p* < 0.001), and is in line with previous research^25^. In addition, this analysis demonstrates how additional variables (DPLI) can be calculated for each of the regions. In this case, DPLI was not found to be sensitive to pressure differences associated with disease duration.

**Table 2:** Results for effect of disease duration on pressure variables.

Region	Variable	Disease duration		*p*-value (95% CI)
		Short	Long	
Forefoot	PP (kPa)	657	1022	< 0.001 [208, 557]
	PTI (kPa/s)	347	678	< 0.001 [226, 454]
	DPLI	0.03	0.06	0.33 [− 0.02, 0.07]
Hindfoot	PP (kPa)	358	391	0.712 [− 104, 129]
	PTI (kPa/s)	145	156	0.98 [− 38, 32]
	DPLI	0.06	0.07	0.75 [− 0.04, 0.04]

### Ethics approval and consent to participate

Ethical approval for this study was obtained from the local National Health Service committee (West of Scotland 4, reference number 14/WS/1150), and participants gave informed, written consent upon enrolment. All methods were performed in accordance with the relevant guidelines and regulations including the ethical principles laid out in the Declaration of Helsinki.

## Discussion

Biomechanical pressure data, in particular for plantar pressures, can provide useful information to researchers and clinicians. Such use cases include assessing areas in the foot that have pain, discomfort, or damage, as well as more esoteric applications like studying genetic factors in twin studies^26^, or measuring pressures in animal gait^27^. We believe pressure distribution measurements can provide many further scientific and clinical insights and thus there is a need for an open and flexible software package to process this type of data.

The pressuRe package has the key benefits of being both reproducible and highly customizable. The open-source nature of R’s base functions and large body of user developed packages allows users to easily make their complete analysis code for their studies available. This helps to provide full transparency and reduces ambiguity caused by the limitations of written descriptions or the need for proprietary software. To be clear, the aim of this package is not to perfectly replicate any particular commercially available tools or masking schemes, but in some cases similar functions are included to allow users to produce and present common analyses in a reproducible manner.

There are some limitations we should note. Not all researchers or other individuals will be R users or be familiar with similar programs. We aim to add further tutorials and vignettes to help encourage more people to use the package. The package still relies on the pressure data being saved in a open, human readable format, which means that legacy datasets saved in proprietary formats cannot be analyzed.

We believe this approach will help facilitate the dissemination of new analysis techniques throughout the research community. We plan to continue to add further functionality to the package, including support for analysis techniques such as statistical parametric analysis^28^ and plantar pressure gradients^29^. Requests for new features and bug reports can be submitted via Github or by contacting the corresponding author directly. As the package matures, we will also be adding to a series of vignettes describing the common analysis workflows. In its current state, the package has been tested against existing software to ensure the validity of the results. However, errors are possible across the 3000+ and growing lines of code that make up the package and we would encourage users to report any issues that they find.

In conclusion, the open-source pressuRe package is designed to provide researchers and other users with tools to process, analyze, and visualize pressure data collected on a range of different hardware systems in a standardized manner. We believe this will help overcome some of the current limitations with lack of reproducibility and aid in the advancement of this field.

### Citation of pressuRe

Researchers using PressuRe in a published paper should cite this article and the version of the package used. Citation information can be obtained by typing at the command prompt:

- > Citation(pressuRe)

### Supplementary Information

Supplementary Information.

## Data Availability

The underlying code and sample data for all functions is available at https://github.com/Telfer/pressuRe. The development version of the package can also be downloaded from here between periodic updates on CRAN.
