# Supplementary material for: pressuRe: an R package for analyzing and visualizing biomechanical pressure distribution data
Source: Sci Rep. 2023 Oct 5;13:16776. doi: 10.1038/s41598-023-44041-6 (PMC10556014; doi:10.1038/s41598-023-44041-6)
Supplement: Supplementary file 1 — Supplementary Information. [file 41598_2023_44041_MOESM1_ESM.pdf]

pressuRe_Vignette

Scott Telfer

2023-08-14

Introduction

This brief document describes a simple approach to processing plantar pressure data from a study on patients with diabetes.

Outline code

packages required

```
library(pressuRe)
library(tidyverse)
```

set data directory

Directory for pressure data. Data is stored in in sub-folders by participant

```
press_dir <- "C:/Users/telfe/OneDrive - UW/CoRE 
Projects/DFOOTPRINT/Pressure_files/"
```

Extract participant ids

```
participants <- list.dirs(press_dir, full.names = FALSE, recursive = FALSE)
```

Analysis

Set up empty data frame to store results

```
barefoot_df <- data.frame(participant = factor(), side = factor(),
                          pressure_variable = factor(),
                          mask_name = factor(), value = double())
```

Simple loop to process data files. This takes a bit of time to run

```
for (prtc in seq_along(participants)) {
  # get file paths and split into left and right
  emed_files <- list.files(paste0(press_dir, participants[prtc]), ".lst",
                           full.names = TRUE)

  for (fn in seq_along(emed_files)) {
    print(paste0(prtc, " ", fn))
    # import data
    pressure_data <- load_emed(emed_files[fn])

    # get side
    if (str_detect(emed_files[fn], "_r") == "TRUE") {
```

```
      side <- "RIGHT"} else {side <- "LEFT"}

    # mask
    pressure_data <- create_mask_auto(pressure_data, "automask_simple",
                                      plot = FALSE)

    # extract variables
    pp <- mask_analysis(pressure_data, FALSE, "press_peak_sensor")
    pti <- mask_analysis(pressure_data, FALSE, "pti_1")
    dpli <- mask_analysis(pressure_data, FALSE, "dpli")

    # add to df
    df <- bind_rows(pp, pti, dpli)
    df <- cbind(participant = as.factor(rep(participants[prtc], nrow(df))),
                side = as.factor(rep(side, nrow(df))), df)
    barefoot_df <- bind_rows(barefoot_df, df)
  }
}
```

Summary results

```
bf_summary <- barefoot_df %>%
  group_by(participant, side, pressure_variable, mask_name) %>%
  summarize(mean_val = mean(value)) %>% filter(mask_name != "midfoot_mask")
```

Statistical analysis

Disease duration data frame

```
disease_duration <- c(19, 20, 18, 1, 10, 2, 33, 11, 49, 20, 5, 15, 7, 19, 30,
4)
dd_df <- data.frame(participant = factor(participants),
                    disease_duration = disease_duration)
dd_df <- dd_df %>% mutate(disease_duration_fact = case_when(disease_duration
< 12 ~ "SHORT",
                                                            disease_duration
>= 12 ~ "LONG"))
dd_df$disease_duration_fact <- as.factor(dd_df$disease_duration_fact)

# add the disease duration data to the bf and inshoe data frames
bf_summary <- left_join(bf_summary, dd_df, by = "participant")
```

Here we are treating each foot as independent, this is likely not a valid assumption but given the small numbers we are working with it's fine for demonstration purposes. We'll use Mann Whitney u test given small numbers

```
#### peak pressure
bf_pp_ff <- bf_summary %>% filter(pressure_variable == "peak_press_sensor")
%>%
  filter(mask_name == "forefoot_mask")
wilcox.test(mean_val ~ disease_duration_fact, data = bf_pp_ff,
```

```
             paired = FALSE, exact = FALSE, conf.int = TRUE)
bf_pp_hf <- bf_summary %>% filter(pressure_variable == "peak_press_sensor")
%>%
  filter(mask_name == "heel_mask")
wilcox.test(mean_val ~ disease_duration_fact, data = bf_pp_hf,
             paired = FALSE, exact = FALSE, conf.int = TRUE)

#### pti
bf_pti_ff <- bf_summary %>% filter(pressure_variable == "pti_novel") %>%
  filter(mask_name == "forefoot_mask")
wilcox.test(mean_val ~ disease_duration_fact, data = bf_pti_ff,
             paired = FALSE, exact = FALSE, conf.int = TRUE)
bf_pti_hf <- bf_summary %>% filter(pressure_variable == "pti_novel") %>%
  filter(mask_name == "heel_mask")
wilcox.test(mean_val ~ disease_duration_fact, data = bf_pti_hf,
             paired = FALSE, exact = FALSE, conf.int = TRUE)

#### dpli
bf_dpli_ff <- bf_summary %>% filter(pressure_variable == "dpli") %>%
  filter(mask_name == "forefoot_mask")
wilcox.test(mean_val ~ disease_duration_fact, data = bf_dpli_ff,
             paired = FALSE, exact = FALSE, conf.int = TRUE)
bf_dpli_hf <- bf_summary %>% filter(pressure_variable == "dpli") %>%
  filter(mask_name == "heel_mask")
wilcox.test(mean_val ~ disease_duration_fact, data = bf_dpli_hf,
             paired = FALSE, exact = FALSE, conf.int = TRUE)
```

Simple linear model treating disease duration as a continuous variable

```
mod <- lm(data = bf_pp_ff, mean_val ~ disease_duration)
summary(mod)
mod <- lm(data = bf_pp_hf, mean_val ~ disease_duration)
summary(mod)
mod <- lm(data = bf_pti_ff, mean_val ~ disease_duration)
summary(mod)
mod <- lm(data = bf_pti_hf, mean_val ~ disease_duration)
summary(mod)
mod <- lm(data = bf_dpli_ff, mean_val ~ disease_duration)
summary(mod)
mod <- lm(data = bf_dpli_hf, mean_val ~ disease_duration)
summary(mod)
```

Plot

```
bf_plot <- bf_summary
levels(bf_plot$pressure_variable) <- c("Peak pressure", "PTI", "DPLI")
levels(bf_plot$mask_name) <- c("Forefoot", "Hindfoot", "Midfoot")
g <- ggplot(bf_plot, aes(disease_duration, mean_val))
g <- g + geom_point()
g <- g + facet_grid(rows = vars(pressure_variable), cols = vars(mask_name),
scales = "free")
```

```r
g <- g + theme_bw() + xlab("Disease duration (years)") + ylab("Pressure variable")
print(g)
```
